# Vascular CT and MRI: a practical guide to imaging protocols

**DOI:** 10.1007/s13244-018-0597-2

**Published:** 2018-03-14

**Authors:** D. J. Murphy, A. Aghayev, M. L. Steigner

**Affiliations:** Cardiovascular Imaging Program, Department of Radiology, Brigham and Women’s Hospital, Boston, MA USA

**Keywords:** Computed tomography angiography, Magnetic resonance imaging, Angiography, Magnetic resonance angiography, Atherosclerosis

## Abstract

Non-invasive cross-sectional imaging techniques play a crucial role in the assessment of the varied manifestations of vascular disease. Vascular imaging encompasses a wide variety of pathology. Designing vascular imaging protocols can be challenging owing to the non-uniform velocity of blood in the aorta, differences in cardiac output between patients, and the effect of different disease states on blood flow. In this review, we provide the rationale behind—and a practical guide to—designing and implementing straightforward vascular computed tomography (CT) and magnetic resonance imaging (MRI) protocols.

Teaching Points

*• There is a wide range of vascular pathologies requiring bespoke imaging protocols.*

*• Variations in cardiac output and non-uniform blood velocity complicate vascular imaging.*

*• Contrast media dose, injection rate and duration affect arterial enhancement in CTA.*

*• Iterative CT reconstruction can improve image quality and reduce radiation dose.*

*• MRA is of particular value when imaging small arteries and venous studies.*

**Electronic supplementary material:**

The online version of this article (10.1007/s13244-018-0597-2) contains supplementary material, which is available to authorised users.

## Introduction

Non-invasive cross-sectional imaging plays a crucial role in the assessment of the varied manifestations of vascular disease, and in both the planning and follow-up of minimally invasive interventional techniques. Designing vascular imaging protocols can be challenging owing to the non-uniform velocity of blood in the aorta, differences in cardiac output between patients and the effect of different disease states on blood flow that cannot be predicted pre-scan. The complexity of the disease under investigation also influences vascular imaging protocols; for example, the need for a delayed phase to detect endoleaks in patients post endovascular aortic aneurysm repair (EVAR). In this review, we endeavour to provide the rationale behind and a practical guide to designing and implementing straightforward vascular computed tomography (CT) and magnetic resonance imaging (MRI) protocols (Tables 1 and 2).

**Table 1:** Sample CT vascular protocols

Protocol	I rate (ml/s)	Bolus tracking level	ECG-gating^b^	Non-contrast	Arterial	Delay (immediate unless specified)
AA I-	–	–	–	A/P	–	–
AA I+	4	Coeliac artery	–	–	A/P	–
AA Post-op stent	4	Coeliac artery	–	A/P	A/P	A/P (70s)
AA Pre-op stent	4	Coeliac artery	–	A/P	A/P	–
Chest venogram^a^	3	–	–	–	–	C (50 & 100 s)
Pulmonary angiogram	4	Main PA	–	–	C	–
TA I+	4	Descending TA	+	C	C	–
TA I-	–	–	+	C	–	–
TA Post-op stent/extravasation	4	Descending TA	–	C	C	C (70 s)
CAP Aorta	4	Descending TA	+	C	C/A/P	–
Lower extremity CTA	5	Renal artery	–	Kidney-toes^b^	Kidney-toes	Knees-toes
Lower extremity CTA + CTV^a^	5	Renal artery	–	Kidney-toes^b^	Kidney-toes	Kidney-toes (180 s)
Re-do sternotomy	5	Descending TA	+	–	C	–
Mesenteric ischaemia	5	Coeliac artery	–	–	A/P	A/P (60 & 90s)
Pelvic vein CTV^a^	3	–	–	–	–	A/P (100 & 180 s)
Renal arteries	5	Coeliac artery	–	–	A	–
Renal donor	5	Coeliac artery	–	A/P	A	A (100 s) & A/P (6 m)
Renal recipient	5	Coeliac artery	–	A/P	A/P	A/P (150 s)
SGAP (prone)	5	Aorta bifurcation	–	–	Umbilicus-knees	–
Upper extremity CTA	5	Arch	–	Arch-fingers^b^	Arch-fingers	Elbow-fingers
DIEP	5	Aorta bifurcation	–	–	A/P	–

Peak kilovoltage (kVp) is chosen at 140/120/100/80 kVp dependent on BMI

Tube current (mA) is determined by automatic exposure control

Tube rotation time is maximum

All phases are reconstructed with a slice thickness of 1 mm at an interval of 0.8 mm and sent to a 3D post-processing workstation

*AA* abdominal aorta, *TA* thoracic aorta, *C* chest, *A* abdomen, *P* pelvis, *I* iodinated contrast, *Arch* aortic arch, *MPA* main pulmonary artery, *CTA* CT angiogram, *CTV* CT venogram, *DIEP* deep inferior epigastic perforators, *SGAP* superior gluteal artery perforators, *ROI* region of interest

All protocols receive 100 ml of iodinated contrast, except for venogram protocols^a^, which receive 125 ml

^b^Optional

**Table 2:** Sample MRI vascular protocols

Protocol	C (mmol/kg)*	Localizer	T2 SSFSE	GRE CINE		Mask + infusion	TR-MRA	CE-MRA (2 phase)	CE- MRA delay	T1FSGRE	HR T1FS GRE ± C	PC
Thoracic aorta	0.1	Chest	Axe + Cor	AV		Sag obl	–	Sag obl	–	Axe	–	–
Abdominal aorta	0.1	Abdo	Axe + Cor	–		Cor	–	Cor	–	Axe	–	–
Dissection	0.1 0.1	Chest Abdo	Axe + Cor Axe + Cor	AV –		Sag obl Cor	– –	Sag obl Cor	– –	Axe Axe	– –	– –
Aortitis	0.1 0.1	Chest Abdo	Axe + Cor Axe + Cor	AV –		Sag obl Cor	– –	Sag obl Cor	– –	– –	Axe Axe	– –
Thoracic outlet	0.1 0.1	Chest (arms up) Chest (arms down)	Axe + Cor + Sag Axe + Cor	– –		Cor Cor	– –	Cor Cor	– –	Axe Axe	– –	– –
Coarctation	0.1	Chest	Axe + Cor	AV		Sag obl	–	Sag obl	–	Axe	–	Axe
Superior vena cava	0.1	Chest	Axe + Cor	–		Cor	–	Cor	Cor	Axe + Cor	–	–
Mesenteric MRA	0.1	Abdo	Axe + Cor	–		Cor	–	Cor	Cor	Axe + Cor	–	–
Renal artery	0.1	Abdo	Axe + Cor	–		Cor	–	Cor	Cor	–	Axe + Cor	
DVT-pelvis	0.1	Abd/Pelvis Thigh	Axe + Cor Cor	– –		Cor –	– –	Cor –	Cor –	Axe + Cor Cor	– –	– –
Pulmonary embolus	0.1	Chest	Axe + Cor	–		Cor	Cor	Cor	–	Axe + Cor	–	–
Upper extremity MRA	5 ml 5 ml 0.1	Hand Forearm Arm/chest	– – Axe + Cor	– – –		– – Cor	Cor Cor –	– – Cor	– – –	Axe Axe Axe + Cor	– – –	– – –
Lower extremity MRA	5 ml 5 ml 0.1	Foot Calf Abdo/pelvis/thigh	– – –	– – –		– – Cor (3 stn)	Sag Cor –	– – Cor (3 Stn)	– – –	– – Axe + Cor	– – –	– – –
Poplitea entrapment^**^	0.1	Knee	Axe + Cor	–		Cor	Cor	Cor	–	Axe + Cor	–	–

**T2 SSFSE** T2-weighted single shot fast spin echo, **GRE** gradient echo, **DIR** double inversion recovery, **TR-MRA** time-resolved MRA, **CE-MRA** T1 spoiled gradient echo contrast-enhanced MRA, **T1FS GRE** T1-weighted 3D spoiled gradient echo sequence with a fat selective prepulse, **HRT1FS GRE ± C** high resolution T1-weighted 3D spoiled gradient echo sequence with a fat selective prepulse pre- and post-contrast, **PC** phase contrast

*C* Gadolinium-based contrast agent, *Abdo* abdomen, *Axe* axial, *Cor* coronal, *Sag* sagittal, *Sag Obl* sagittal oblique, *AV* aortic valve, *Stn* station

^a^Contrast dose is expressed in mmol/kg unless otherwise specified and is injected at a rate of 1.5 ml/s followed by a saline chaser

^b^Protocol is performed with the feet in the neutral, dorsiflexed and plantarflexed positions

## Technical

### Computed tomography angiography (CTA)

CT is a quick, non-invasive imaging modality with excellent spatial and temporal resolution. Modern CT scanners can provide sub-millimetre isotropic three-dimensional (3D) datasets within a single breath-hold during the first past of intravenous (IV) iodinated contrast medium (CM). One of the minimum requirements for more advanced CTA applications, such as coronary CTA, is a 64-channel CT; for many of the other less challenging vascular CT protocols, such as abdominal aorta or visceral aneurysm assessment, a 16-channel CT is adequate. The continued evolution of CT technology is based in no small part on the demands that cardiovascular imaging places in terms of speed, temporal resolution and scan volume. To help cope with the demands of cardiovascular imaging, manufacturers have made significant improvements in *z*-axis volume coverage, detector and tube technology, with different emphasis depending on the vendor [1]. State of the art wide-area detector CT scanners, with up to 320-detector rows, can provide up to 16-cm *z*-axis detector coverage in a single gantry rotation; this allows for large volume coverage in both helical and axial (step and shoot) acquisition modes [2]. Dual-source CT scanners provide the maximum temporal resolution available, as the temporal resolution is equal to a quarter of the gantry rotation time; this is as low as 66 milliseconds (ms) in the third-generation scanners. Maximising temporal resolution is advantageous when imaging structures prone to cardiac motion artefact, such as the aortic root [3], or when imaging patients prone to motion, such as trauma patients or poor breath-holders.

Obtaining satisfactory arterial enhancement is crucial in the assessment of intravascular pathology. One of the important scan parameters that can influence arterial enhancement is scan acquisition time. A short acquisition time is preferable once the scan begins (in most situations) to ensure uniform arterial opacification on the acquired images. For helical scans, the acquisition time is equal to the gantry rotation time multiplied by the number of gantry rotations required to cover the anatomical area. The number of gantry rotations is determined by the scan range divided by the product of the detector bank width and the pitch. Axial scanning acquires multiple volumes, and is used in particular when performing ECG-gated studies such as coronary and aortic CTA, to help reduce radiation dose [4]. For axial acquisitions of volumes smaller than the width of detectors, the scan time is equal to the gantry rotation time. When axial scanning is used for volumes larger than the detector width, the total scan time is equal to the total number of volumes required to cover the desired anatomical area multiplied by the gantry rotation time, added to the sum of the interscan time intervals required for table repositioning.

Optimising IV contrast medium (CM) administration is important in obtaining strong arterial enhancement during CTA. The degree of enhancement of a system is proportionally related to the concentration of iodine within it. There is variation in the relationship between enhancement and iodine concentration in different CT scanners, but it is the range of approximately 25-30 Hounsfield units (HU) per milligram (mg) of iodine per millilitre (ml) at 120 peak kilovoltage (kV) [5]. The easily adjustable factors that determine arterial enhancement in CTA are the concentration of iodine in the CM used, the injection rate and the injection duration.

There is an almost linear relationship between enhancement and iodine concentration, which makes CM preparations with a high iodine concentration ideal (preferably 350-400 mg/ml) for CTA when the injection rate is fixed, resulting in a higher iodine delivery rate. The concept of iodine delivery rate (IDR, mg/s) is a method of standardising the rate of iodine delivery across CM with different iodine concentrations, and is calculated from the following formula: IDR = [CM iodine concentration (mg/ml)/1,000] × flow rate (ml/s) [6]. High iodine delivery rates are important in providing diagnostic image quality in CTA [7]. If CM preparations with lower concentrations of iodine are used, for example 300 mg/ml, the injection parameters can be adjusted to ensure a similar iodine delivery rate to that of a higher iodine concentration CM preparation[8]; for example, in a porcine model undergoing CT pulmonary angiography, CM with an iodine concentration of 300 mg/ml injected at a rate of 5 ml/s provided an identical IDR of 1.5 g/s to 370 mg/ml CM injected at a rate of 4.1 ml/s [9]. CM with lower iodine concentrations have lower viscosity, with reduced injection pressures, which may potentially reduce extravasation risk [6, 8]; however, for a fixed scan duration, the higher injection rate required to keep the same iodine delivery rate would result in an increase in total CM volume required [6]. Experimental models suggest that an IDR of 1.5-2 g/s provides adequate arterial opacification (>200 HU) in CTA protocols, regardless of the concentration of CM used [10, 11].

The strength of arterial enhancement (peak HU) is proportional to the injection rate, and the duration of enhancement to the injection length [12]. Increasing the injection rate leads to a faster accumulation of contrast in the aorta, increasing peak aortic enhancement. For a fixed contrast volume, however, this reduces injection duration, and in turn reduces the available time window to acquire the CT within. With modern scanners, an injection rate of 4-5 ml/s is usually sufficient in providing excellent arterial opacification for most vascular studies; venous imaging does not require as high injection rates. The traditional approach to determine injection duration was to match it to scan duration time; however, with modern multi-detector, fast CT scanners, this may result in inadequate opacification due to a lower volume of CM being delivered. One approach for estimating injection duration is to set a minimum duration of 10 s, and to add on the estimated scan duration time, which is available from all vendors after the scan range has been chosen. CTA requires use of a power-injector to allow uniform high injection rate CM bolus delivery. Use of a saline flush should be routine to help push the tail of the CM bolus into the central blood volume, as without it, the bolus tail would remain unused in the peripheral veins [13]. The saline chaser also helps reduce intravascular CM dispersion and reduces streak artefact from dense contrast in the brachiocephalic veins and superior vena cava, which is especially important in thoracic CTAs [14].

Obtaining high-quality arterial enhancement depends on various CT scanner, CM and patient-related factors. Even when CM and scanner use remain constant, patient factors such as body size, cardiac output and disease state can influence inter-individual variation in arterial enhancement. For example, large calibre and diseased vessels may take longer to opacify than normal. Reduction in cardiac output means that the CM bolus is slower to arrive and clear, resulting in delayed, but stronger peak arterial enhancement. These differences mean that the same scan timing delay cannot be used for everyone, and it needs to be tailored to the individual. Two methods commonly used to provide accurate CTA scan timing are the test bolus and bolus tracking methods. The test bolus method is based on injecting a small quantity (10-20 ml) of CM, then obtaining multiple low radiation dose images at a fixed time interval. By placing a region of interest (ROI) over the target vessel, a time-enhancement curve can be plotted to determine the time to peak enhancement. This can then be used to estimate the scan delay for the CTA. The bolus tracking technique involves acquiring a pre-contrast image at a reference level with placement of an ROI over a target vessel. After the CM injection is started, a low-dose monitoring scan is performed at a predetermined level after a fixed time delay, usually 5 s, and thereafter every 1-3 s until the enhancement in the ROI reaches a specified level (typically 150 HU). The CTA then begins after a pre-specified adjustable delay to allow peak arterial enhancement (approximately 8 s); this delay must also take into account time for table repositioning. The two methods are comparable in terms of satisfactory CTA timing, with bolus-tracking frequently used due to its reduced examination time and ease of use [15]. The test bolus method is useful in patients with challenging anatomy, such as congenital heart disease patients post complex surgical repair [16, 17].

The role of CM as a causative agent in acute kidney injury (AKI) is currently a topic of debate, with recent studies suggesting the risk of contrast-induced nephropathy (CIN) may not be as high as previously thought [18–20]. The use of CM in patients with normal renal function is safe, with no evidence of a significant drop in glomerular filtration rate (GFR) post CM administration [21]. To mitigate against the possible risk of CIN, CM should only be given to patients with severe renal dysfunction (GFR <30 ml/min) or AKI on a case-by-case basis after a risk-benefit analysis [22–24]. There is no evidence available that reducing CM volume in patients with mild-to-moderate renal impairment (GFR 60-30 ml/min) has an effect on development of CIN.

Many modern scanners automate peak kilovoltage (kV) selection based on the topogram. Reducing the kV, for example from 120 to 100 in patients with a body mass index (BMI) of <25, can help improve image quality, and may potentially reduce radiation dose [25]. Much of the radiation dose reduction achieved by reducing kV is offset in the presence of automatic exposure control (AEC), which increases the tube current to maintain a user-specified noise level [26]; for example, to maintain diagnostic image quality, the tube current approximately doubles for a reduction from 120 to 100 kV [27]. Small radiation dose reductions are still achievable with the use of model-based iterative reconstruction techniques [28], but most of the benefit from reduced kV scanning in the presence of AEC in CTA comes from improved vessel contrast. Lower kV CTA has higher vessel HU values due to relatively increased attenuation of iodine as the kV nears its k-edge of 33 kV, improving image signal to noise and contrast to noise ratios [26]. Lower injection rates should be used in reduced kV CTA (for example, 4 ml/s at 100 kV, 3 ml/s at 80 kV) to prevent the vessels appearing too high density, like bone; this has the added benefit of reducing the overall volume of CM required [10, 29, 30]. There is, however, a cost to low kV CTA: the higher tube current required to reduce noise requires a larger focal spot, reducing spatial resolution [31]. Blooming artefact from calcified atherosclerotic plaque or metal stents is also exaggerated at lower kV, which can be problematic in CTA interpretation [4].

Dual-energy CT (DECT) is a state-of-the art technology that can improve image contrast in CTA by providing monoenergetic lower-energy reconstructions closer to the k-edge of iodine, with improved image contrast by a relatively increased contribution of the photoelectric effect [32–35]. This can be accomplished using dual-source dual-energy (DSDE) CT systems that employ two separate X-ray tubes situated 90° apart that can operate at two different voltages, single-source dual-energy (SSDE) CT systems with fast kV switching or with single-source CT systems with a dual-layer of detectors [36]. Low-energy monoenergetic reconstructions in CTAs with suboptimal vessel opacification can improve iodine attenuation to levels similar to conventional polyenergetic images obtained with higher volumes of contrast, allowing ‘rescuing’ of a suboptimal CTA [37]; this also has the potential to reduce the iodine load required to obtain a diagnostic CTA, allowing the use of reduced concentration CM preparations and/or a lower volume [38]. Virtual monoenergetic datasets reconstructed at a high kV can help reduce blooming artefact, allowing improved assessment of vascular stent patency [39], and of heavily calcified vessels [40]. DECT allows reconstruction of virtual non-contrast images from post-contrast CT acquisitions by excluding iodine-containing pixels, thus enhancing water attenuation [36]; this has the potential to reduce radiation dose in multiphase vascular CT protocols, by obviating the need to acquire a separate non-contrast CT [41, 42]. DECT can provide an assessment of organ perfusion using iodine map imaging. This is often presented using a colour look-up table, and can improve the detection of embolic disease by detecting areas of parenchymal hypoperfusion; this technique has been shown to improve the diagnostic accuracy of CTA in the detection of pulmonary emboli [43, 44].

Cardiac motion artefact can be problematic when assessing the aortic root, and the use of ECG-gating in thoracic aorta CTA can help to address this. Motion artefact at the aortic root is dependent on several factors; chief among them, the gantry rotation time of the CT scanner and the patient’s heart rate [4]. ECG-gating can help to reduce the ill-effects of cardiac motion on the aortic root, but it does not eliminate it. Pre-scan beta-blockade is another step that can help to reduce motion artefact, and is commonly used in coronary CTA; in practice, the administration of beta-blockers to patients undergoing routine thoracic aorta CTA is not often necessary to obtain satisfactory image quality, particularly with the improved temporal resolution of modern CTs [45]. The available ECG-gating techniques include prospective, retrospective or high pitch gating, with the optimum choice largely scanner dependent [1]. Scanners with large banks of detectors can cover the thorax quickly, making prospective gating ideal. Smaller detector-width scanners are more suited to retrospective gating with tube current modulation. High pitch gated acquisitions are suited to dual-source scanners. The use of ECG-gating does increase radiation dose, primarily determined by the number of cardiac phases, rather than the type of ECG-gating used. The optimum phase (percent of the R-R interval) for image acquisition is heart-rate dependent [46]. When the heart rate is less than 75 beats per minute (bpm), a diastolic acquisition window of approximately 70-80% is preferred, with a systolic phase acquisition (30-40%) used in patients with higher heart rates.

There is no single best ‘one size fits all’ CTA protocol. Depending on the specific indication, it may be useful to obtain a non-contrast phase first; this can be of particular use when assessing calcified plaque, in postoperative patients, and in cases of suspected active haemorrhage. One approach described for a 64-channel CTA is to: (1) fix the scan duration to 10s for all CTAs; (2) adjust the pitch depending on the volume of coverage required; (3) fix the injection duration to 18 s; (4) operate a constant scan delay time of 8 s after CM arrival; (5) adjust the injection rate according to patient weight (5.0 ml/s for a 75-kg patient, ±0.5 ml/s for every 10 kg of body weight) [27]. With this protocol, the long injection duration, combined with the extra 8 s delay post CM arrival, allows adequate time for arterial filling in nearly every patient. A delayed phase may also be helpful depending on the indication, with the timing measured from the end of the CM injection.

### Magnetic resonance angiography (MRA)

MRA is a multiparametric imaging modality, with excellent contrast resolution. Contrast-enhanced MRA (CE-MRA) involves the administration of a gadolinium based contrast agent (GBCA), which shortens blood longitudinal relaxation (T1). A rapid 3D T1-weighted spoiled gradient echo (GRE) pulse sequence with a short repetition time (TR) and echo time (TE) is ideal for CE-MRA. This provides images with high signal-to-noise ratio (SNR), good spatial resolution and is free from flow-related artefacts [47]. Subtraction techniques improve contrast resolution in CE-MRA. This reduces signal from background tissues by acquiring a mask image prior to GBCA injection, and subtracting it from the post-contrast imaging.

In general, an injection rate of 1.5 ml/s provides arterial imaging with high vessel to background contrast; this can be improved by increasing the injection rate, but similar to CTA, this reduces the available time window to acquire the scan for a fixed volume of contrast. Two methods are commonly used to appropriately time CE-MRA imaging. Similar to the method described for CTA, a test bolus method can be performed, administering 1-2 ml of GBCA and acquiring a series of rapid two-dimensional (2D) images of the vessel in question to determine the optimum time to start imaging post injection. In fluoroscopic triggering, the full bolus of contrast is administered and fluoroscopic-like images of the area of interest are obtained, and when the bolus is detected within the vessel, the technologist can trigger scan acquisition.

Two different acquisition modes are common in CE-MRA, single phase and time-resolved MRA. Single phase MRA captures vascular images at a single point in time. Time-resolved MRA consists of multiple acquisitions of an imaged volume over successive time points post GBCA administration. It is often known under vendor-specific acronyms such as TWIST (Siemens, Erlangen, Germany), TRICKS (General Electric, Chicago, IL, USA), 4D-TRAK (Philips, Best, Netherlands), TRAQ (Hitachi, Tokyo, Japan) and Freeze Frame (Toshiba, Otawara, Japan). This technique is particularly useful in displaying the passage of the contrast bolus through smaller vessels, such as the hands and feet. The core of time-resolved MRA is a 3D–spoiled GRE sequence employing k-space filling tricks to quicken image acquisition, such as non-Cartesian k-space filling, oversampling the centre of k-space (responsible for image contrast) and under sampling of the periphery (responsible for spatial resolution) [48]. These techniques, aligned with the use of parallel imaging, delivers ultra-fast imaging [49].

Paramagnetic contrast agents shorten the T1 and T2 relaxation times of water protons in their immediate surroundings, creating a locally increased magnetic field strength. This change in the local magnetic field strength results in increased local field inhomogeneity, driving the shortening of T1 and T2 relaxation. The resultant increased signal intensity (SI) on T1-weighted images provides the basis behind the use of contrast agents in MR. GBCAs are the most commonly used in MRA, and there are currently nine available GBCAs licensed by the European Medicines Agency (EMA) and the Food and Drug Administration (FDA) in the USA for clinical use. GBCAs can be divided into two different groups, linear and macrocyclic, based on how the ligand chelates the gadolinium ion [50]. In the linear agents, the ligand wraps around the gadolinium (Gd^3+^) ion, but does not completely enclose it. The macrocyclic agents consist of a chelator, which completely surrounds the Gd^3+^ ion in a cage-like structure. The latter agents demonstrate greater stability in vivo than the linear agents, with little (if any) free Gd^3+^ ion dissociation, reducing the risk of nephrogenic systemic fibrosis (NSF) [51, 52]. The recent discovery of cerebral gadolinium deposition in patients with normal renal function is also of concern, although the clinical significance of this phenomenon is yet to be determined [53]. Linear GBCAs are thought to confer a higher risk of cerebral deposition, and their use is now discouraged by the EMA [54], although this guidance has not been reciprocated by the FDA [55]. Cerebral gadolinium deposition is not only associated with linear GBCA use, however, and has been demonstrated in macrocyclic GBCAs in both animals [56] and humans, in particular the macrocyclic agent gadobutrol [57].

The majority of GBCAs in routine clinical use are extracellular fluid (ECF) agents. After injection, they initially distribute in the intravascular space, before rapidly diffusing across the vascular membranes into the interstitial space, eventually establishing an equilibrium between the intravascular and interstitial compartments after approximately 10 min. ECF GBCA agents that demonstrate weak plasma protein binding, such as gadobenate dimeglumine (Gd-BOPTA), help increase relaxivity compared to the other ECF GBCAs [58]. Gadofosveset trisodium (Gd-DTPA-DO3A/MS-325) is currently the only intravascular GBCA licensed by the FDA. It was licensed by the European Medicines Agency for distribution in the European Union (EU) in 2005, but was voluntarily withdrawn from commercial use in the EU by the manufacturer in 2011. It is a linear ionic agent, which binds strongly to albumin, limiting its diffusion into the extravascular space [59].

GBCAs with high relaxivity that remain within the blood-pool are the most attractive from an image quality point of view, however, safety considerations should be taken into account when choosing an agent. Gadobenate dimeglumine has the highest relaxivity of the ECF GBCAs, and gadofosveset trisodium is the only intravascular GBCA. These are both linear ionic agents, placing them in the intermediate risk category for NSF in susceptible patients, and at potentially higher risk for cerebral deposition. The current commercially available GBCA with the most favourable risk profile in terms of NSF and cerebral deposition is the macrocyclic ionic agent gadoterate meglumine. This agent has inferior protein binding and relaxivity compared with some of the other GBCAs, but this is offset by its safety profile. There have to-date been no reported cases of NSF with this agent, even in patients with severe renal dysfunction [51, 52], and it is also the only GBCA in which cerebral deposition has not been demonstrated [56].

For routine MRAs, a straightforward protocol is to begin with localisers of the anatomical area in question, followed by coronal and axial T2-weighted single-shot fast spin echo sequences, which allow a global anatomic assessment. This is then followed with 3D CE-MRA with two successive arterial phase acquisitions, providing anisotropic images, which allows reconstruction of the dataset on a 3D workstation. Finally, an axial T1-weighted 3D spoiled GRE sequence with a fat selective prepulse of the anatomical area can be acquired, allowing for an assessment for significant incidental findings. This basic MRA protocol can then be modified/added to according to the clinical question, as outlined in the anatomical site-specific sections below. It is our practice to administer weight-based GBCA dosing to all patients with GFR >30 ml/min, followed by a saline chaser (Table 2). There is no evidence available to support GBCA dose reduction in patients with mild to moderate renal impairment (GFR 60-30 ml/min). The risk of NSF remains in patients with severe renal dysfunction (GFR <30 ml/min); in these patients the decision to administer GBCA should be made on a case-by-case basis [60].

For patients who cannot have gadolinium, such as those with severe renal impairment (GFR <30 ml/min), non-contrast imaging of the aorta and larger vessels can be performed. In these circumstances, a bright blood imaging, such as a balanced steady state free precession (SSFP) sequence can be used; this is a coherent gradient echo sequence that provides bright blood imaging without gadolinium. In the setting of acute aortic syndromes, this can detect the presence of an aortic dissection with high accuracy when compared with CE-MRA [61, 62]. The major disadvantage of this sequence, however, is the presence of off-resonance artefact; this artefact is more pronounced at higher magnetic field strengths [63]. To overcome this, where possible we perform non-contrast MRAs on 1.5 T rather than at 3.0 T.

### CTA and MRA post-processing

**Fig. 1:**
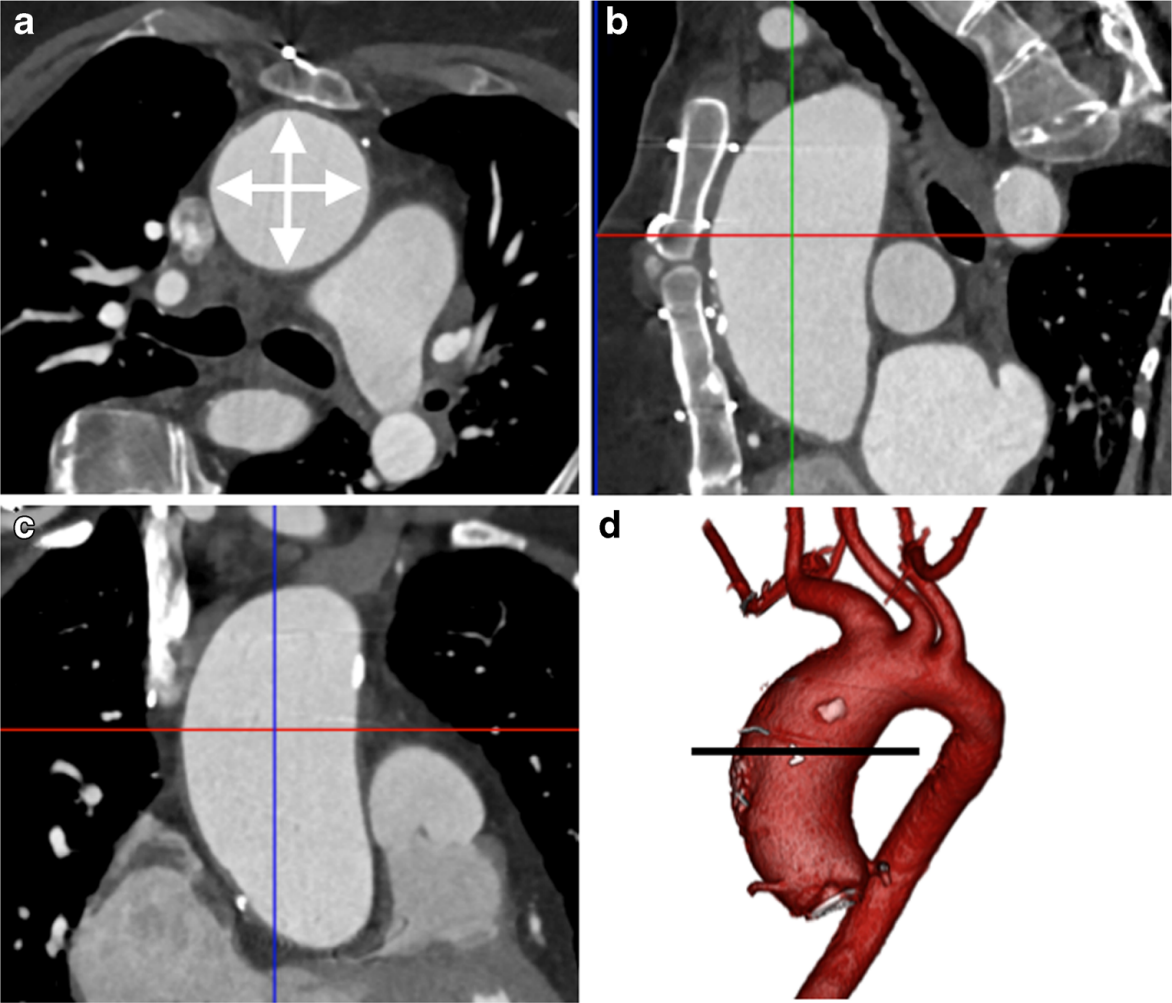
A 45-year-old man with Marfan’s syndrome post aortic valve replacement with a fusiform ascending aortic aneurysm. Measurement of ascending thoracic aorta dimension (*double arrows*) in an axial plane (**a**) will yield erroneous values due to oblique orientation relative to the centerline of the aorta, as demonstrated on sagittal (*red line*, **b**) and coronal (*red line*, **c**) multiplanar reformats (MPRs). Three-dimensional segmented volume rendered (VR) image of the thoracic aorta (**d**) demonstrates the site of measurement (*line*)

**Fig. 2:**
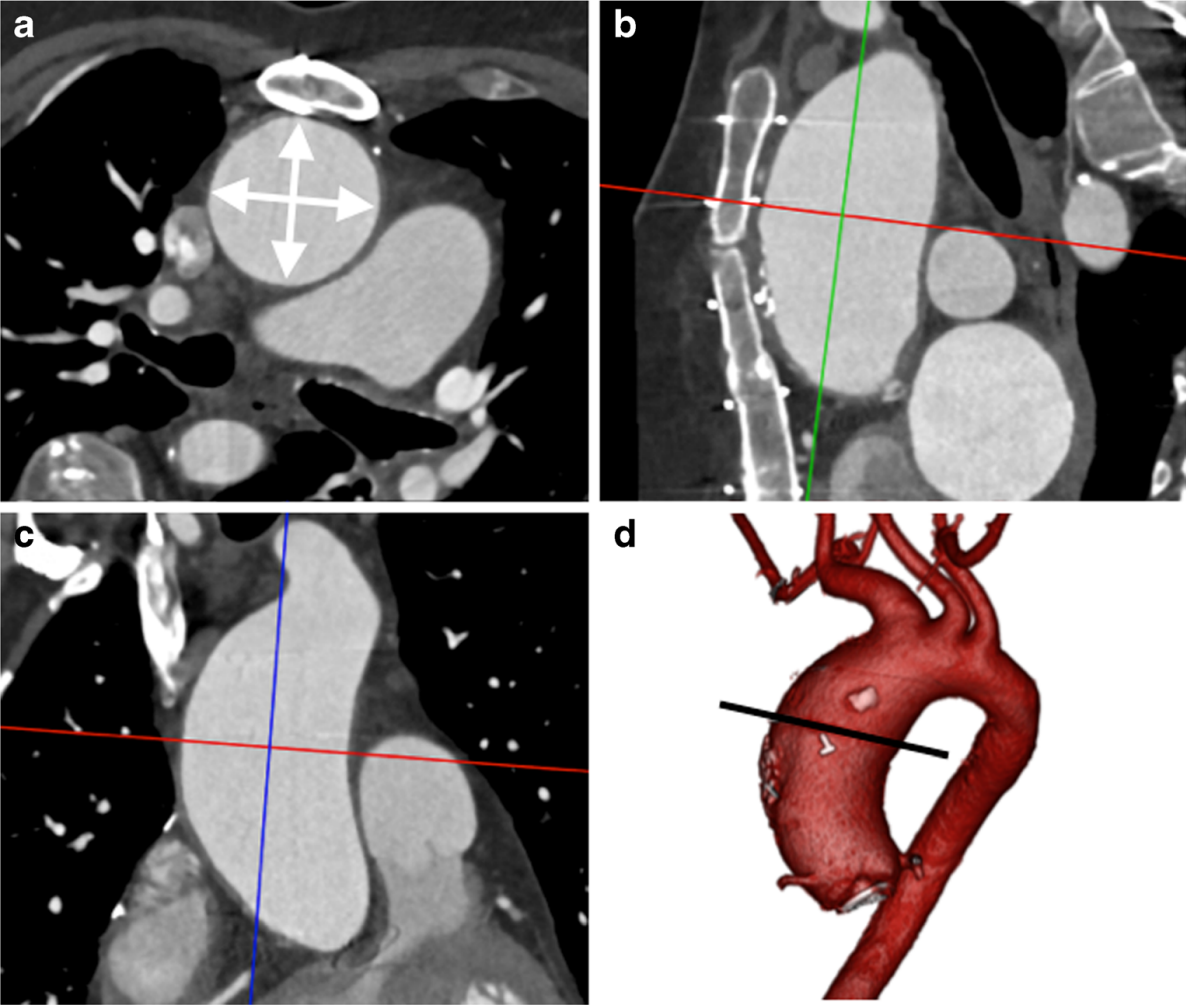
A 45-year-old man with Marfan’s syndrome post aortic valve replacement with a fusiform ascending aortic aneurysm. Measurement of the ascending thoracic aorta dimension (*double arrows*) in a plane double oblique to the vessel centerline (**a**) with sagittal (**b**) and coronal (**c**) MPRs demonstrating the plane of measurement (*red lines*). Three-dimensional segmented VR image of the thoracic aorta (**d**) demonstrates the site of measurement (*line*)

In order to obtain the spatial resolution required, vascular imaging techniques tend to produce large datasets, which can be intimidating and difficult to negotiate. A minimum requirement of any post-processing software package is the ability to perform multiplanar reformats (MPR) of 3D CT or MRI datasets to create 2D images in coronal, sagittal, oblique or curved planes [64]. As the course of vessels tends to not follow along anatomical axial, coronal or sagittal planes; this hinders accurate measurement. Routine use of MPRs to perform measurements in a plane short axis to the centerline of a vessel is the most reliable and reproducible method of performing measurements (Figs. 1 and 2, ESM 1) [65]. The use of semi-automated tools to determine the vessel centerline improves measurement time [66].

Maximum intensity projection (MIP) reconstruction is an algorithm that selects and displays only the voxels with the highest HU (CT) or SI (MRI) of a selected slab in the imaged plane [67]. MIPs allow a global assessment of the imaged vasculature, and are useful in the rapid detection of vascular stenosis and occlusion. Readers must, however, be aware of limitations with this technique such as the overestimation of stenosis due to calcified plaque on CT, and findings should be confirmed with the thin-slice raw data [68].

**Fig. 3:**
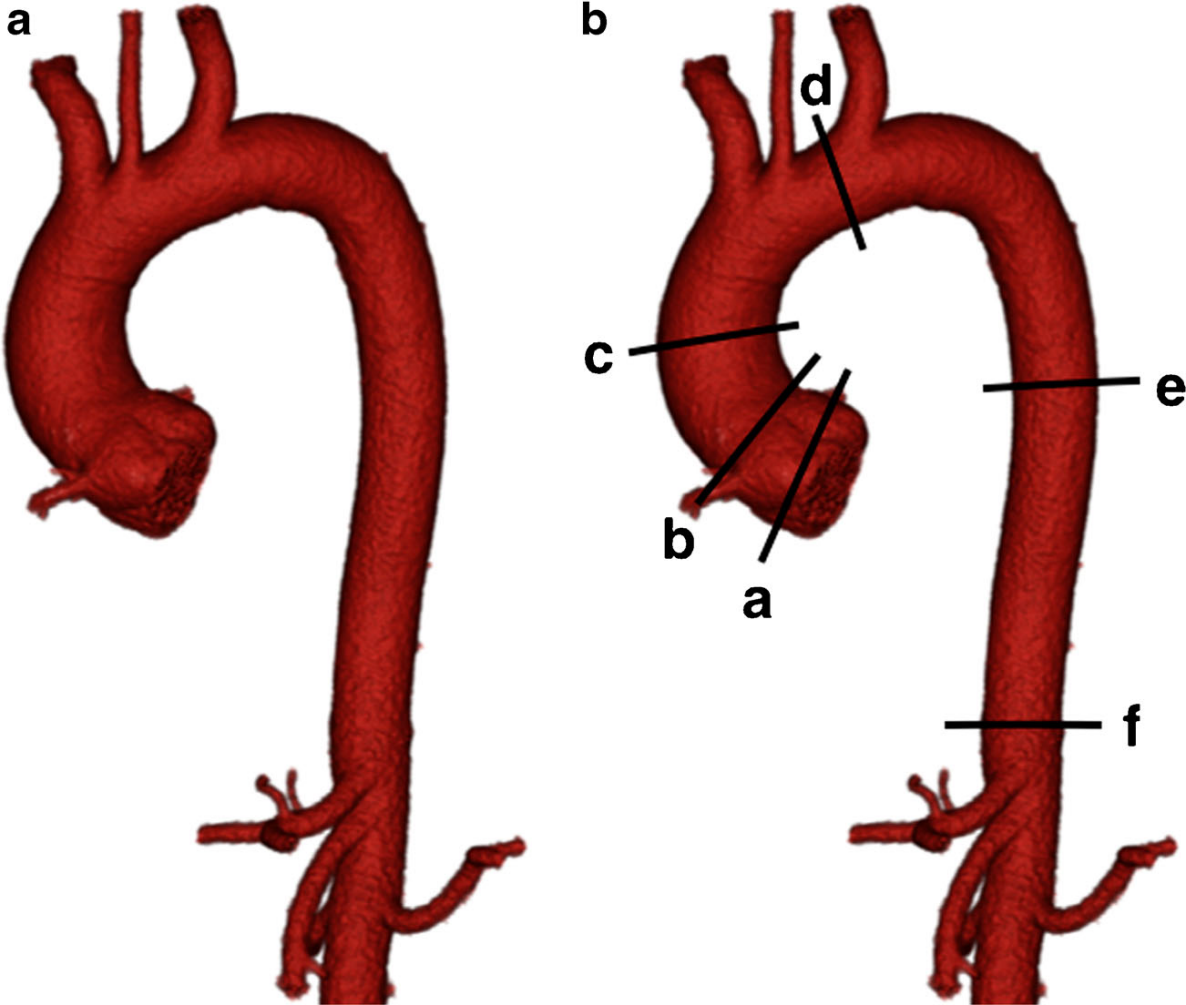
**a** Three-dimensional segmented volume rendered (VR) image of the thoracic aorta with **b** standard sites of measurement. *a* Sinuses of Valsalva, *b* sinotubular junction, *c* ascending thoracic aorta, *d* aortic arch, between origin of left common carotid and left subclavian arteries, *e* descending thoracic aorta, *f* aortic hiatus

Segmented volume-rendered (VR) images can be created using most modern post-processing software packages. Volume rendering operates by assigning opacity values to image data on a scale from 0 to 100% along an artificial line of sight projection [67–69]. VR images are visually attractive, can be useful in pre-procedure planning and are an excellent means of displaying complex anatomy, especially to clinicians with varying knowledge of cross-sectional anatomy (Figs. 3, ESM 2 and 4, ESM 3) [70].

**Fig. 4:**
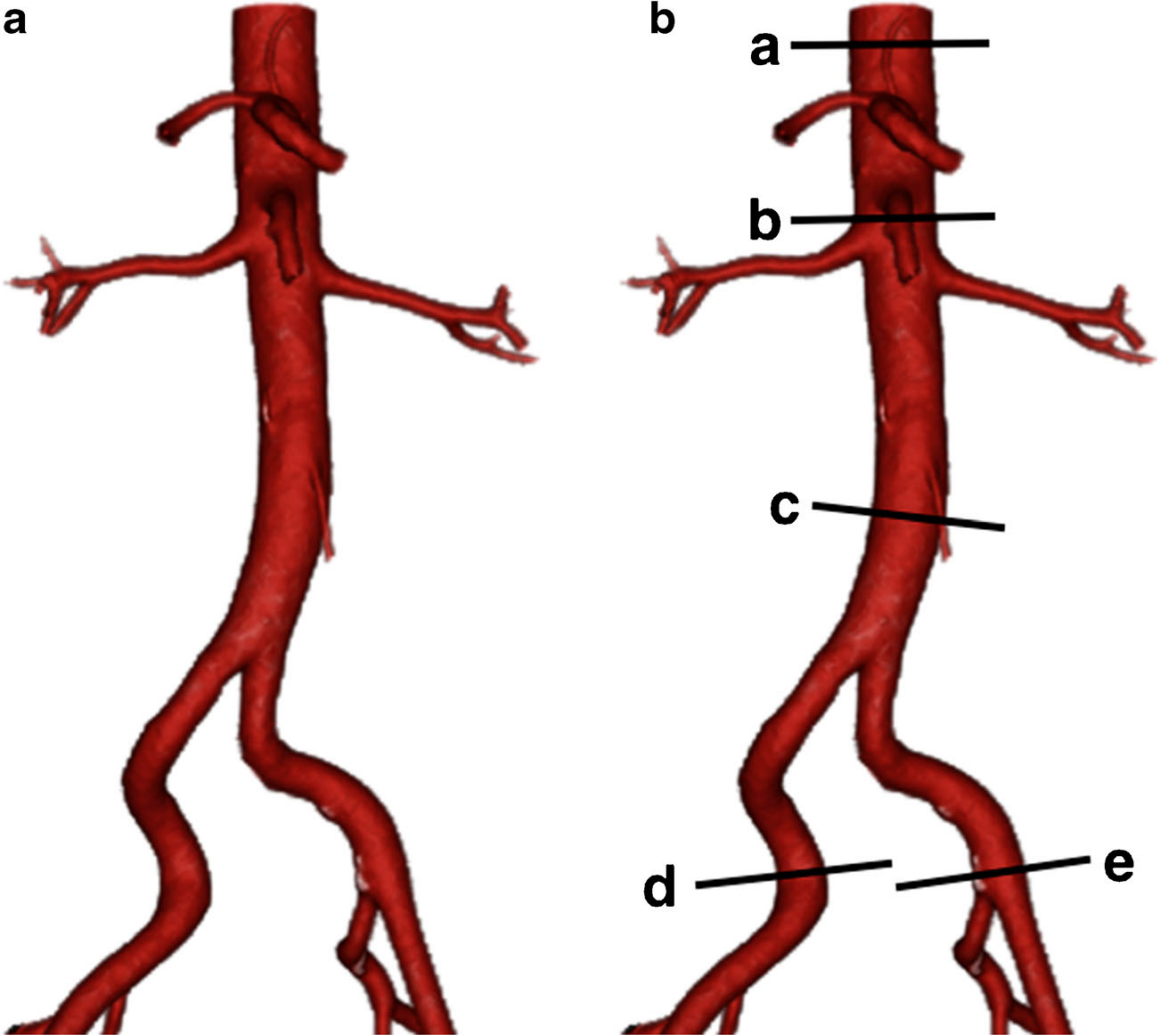
**a** Three-dimensional segmented VR image of the abdominal aorta with **b** standard sites of measurement. *a* Proximal abdominal aorta, *b* juxtarenal abdominal aorta, *c* infrarenal abdominal aorta, *d* right common iliac artery, *e* left common iliac artery

## Notes on specific protocols

### Aorta

**Fig. 5:**
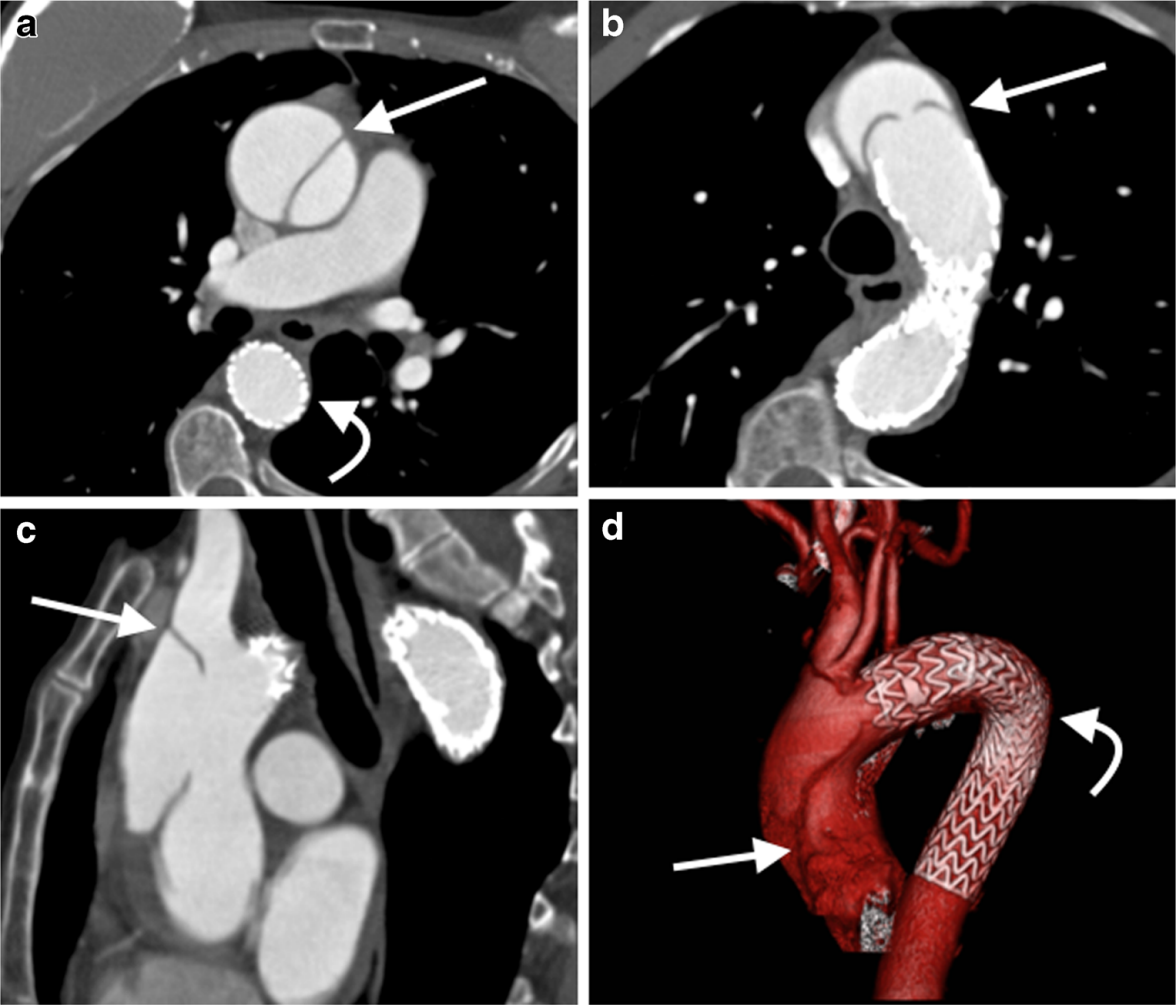
Axial (**a, b**) and sagittal (**c**) arterial phase images from an ECG-gated CT thoracic aorta angiogram in a 62-year-old woman with chest pain demonstrates a type A dissection in the ascending thoracic aorta (**a-c**, *arrow*) with evidence of prior stent graft repair of the descending thoracic aorta (**a-c**, *curved arrow*). Three-dimensional segmented VR image of the thoracic aorta (**d**) delineates the ascending aorta dissection flap (*arrow*) and descending thoracic aorta stent graft (*curved arrow*)

**Fig. 6:**
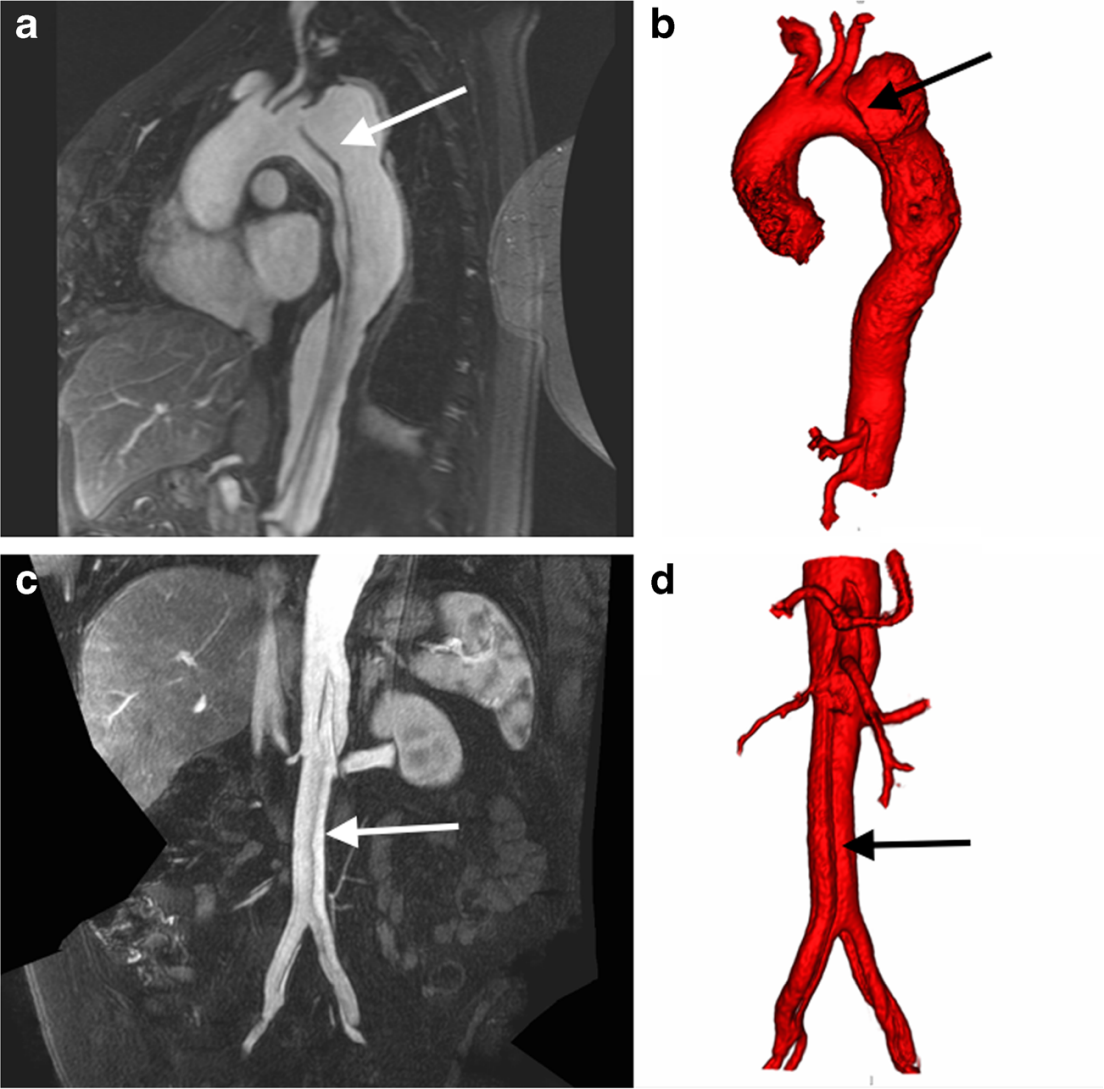
A MR angiogram in a 45-year-old woman who presented with acute onset of tearing chest pain. Sagittal oblique image from a ECG gated T1-weighted post-gadolinium 3D acquisition (**a**) demonstrates a dissection flap (*arrow*) arising in the aortic arch distal to the origin of left subclavian artery consistent with a type B aortic dissection, with the dissection flap extending into the abdominal aorta. Corresponding 3D segmented volume rendered image of the thoracic aorta (**b**) from the thoracic MRA demonstrates the dissection flap (*arrow*). Coronal abdominal T1-weighted post gadolinium MRA image (**c**) demonstrates the distal extent of the dissection flap (*arrow*) into the common iliac arteries bilaterally, with a 3D segmented volume rendered image of the abdominal aorta (**d**) demonstrating the dissection flap in the abdominal aorta (*arrow*)

**Fig. 7:**
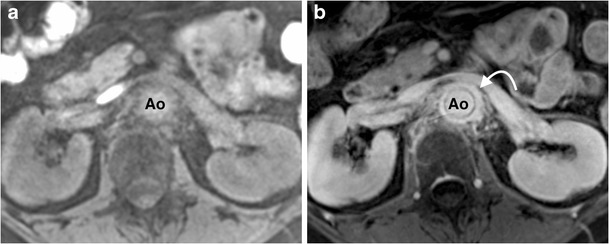
Selected axial images from T1-weighted 3D spoiled gradient echo sequence with a fat selective prepulse sequence pre- (**a**) and post- (**b**) gadolinium in a 55-year-old woman with giant cell arteritis demonstrates enhancing circumferential mural aortic soft thickening (*arrow*) of the juxtarenal aorta (*Ao*) consistent with a large vessel vasculitis

In the emergency setting, imaging of the aorta is primarily focused on the assessment of the acute aortic syndrome (dissection, intramural haematoma or penetrating atherosclerotic ulcer) or for active haemorrhage, and CT is generally the preferred modality (Figs. 3, 4 and 5). Common elective indications for thoracic or abdominal aorta imaging includes the assessment and follow-up of aortic aneurysms, pre- and post-procedure assessment of endovascular aneurysm repair (EVAR) and in the setting of suspected aortitis. Aorta MRA can be used to assess thoracic aortic aneurysms, is the preferred modality in cases of suspected aortitis and aortic coarctation, and can be used to image acute aortic syndromes in selected cases, for example those with a severe iodinated contrast allergy (Figs. 6 and 7).

Acquiring a non-contrast CT prior to aorta CTA is useful to look for high-density intramural haematoma, which can be difficult to visualise after contrast administration. In the post-operative setting, it helps distinguish high-density surgical material such as felt pledgets routinely from a pseudoaneurysm [71–73]. Using ECG-gating in thoracic aorta CTA helps to overcome the effect of cardiac motion on the aortic root, which can hide or mimic significant pathology, such as aortic dissection.

**Fig. 8:**
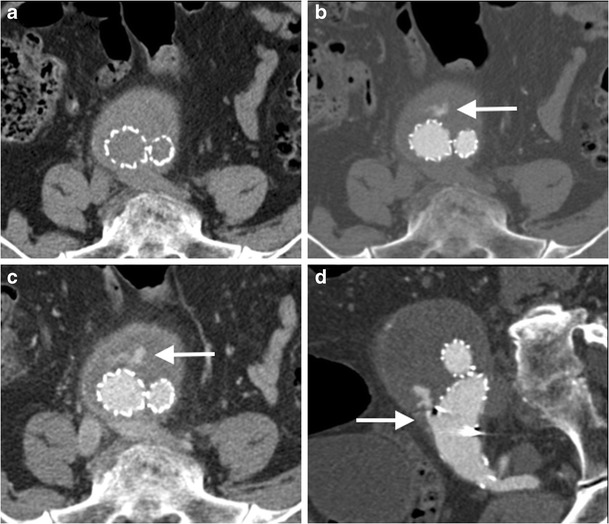
Selected images from a CT angiogram in a 55-year-old man undergoing imaging surveillance post abdominal aortic aneurysm endovascular stent graft repair (EVAR). Axial non-contrast (**a**), arterial phase (**b**) and 70 s delayed phase (**c**) images demonstrate iodinated contrast material within the excluded aneurysm sac (*arrow*) consistent with an endoleak. Sagittal oblique MPR of the right common iliac artery (**d**) demonstrates the endoleak arising from the distal insertion point of the right iliac limb of the EVAR consistent with a type 1b endoleak

**Fig. 9:**
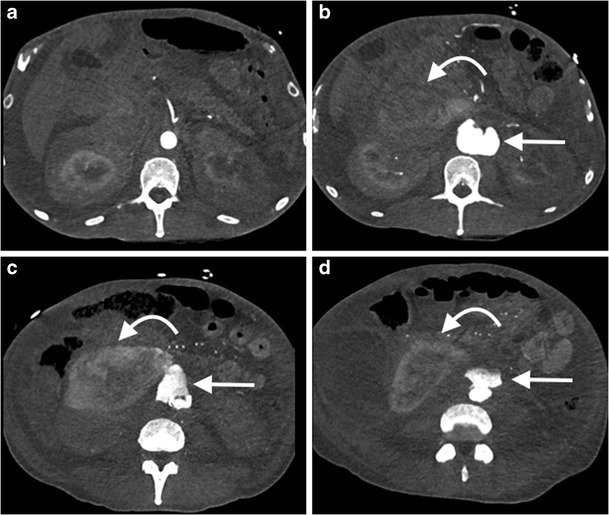
Selected axial images (**a-d**) from an arterial phase CT angiogram of the abdominal aorta in a 55-year-old man with hypotension and abdominal pain demonstrates a large retroperitoneal haematoma (*curved arrow*) and active extravasation from the infrarenal abdominal aorta (*arrow*) consistent with aortic rupture. A ruptured mycotic aneurysm was found in the operating room

For patients undergoing consideration for EVAR, an additional non-contrast CT of the abdomen and pelvis can help delineate calcified plaque (CT AA pre-EVAR, Table 1). Following aneurysm endovascular stent graft repair (EVAR), a CTA with additional 70 s delayed phase imaging (Table 1) can assess both the size of the excluded aneurysm and for the presence of an endoleak (Fig. 8) [74, 75]. This identical protocol can also be used to assess for the presence of active bleeding, with the arterial and delayed phases demonstrating active extravasation, and can help triage patients for IR embolisation (Fig. 9) [76, 77]. When performing multiphasic aortic CTA using a DECT system, reconstruction of a virtual non-contrast image may obviate the need to acquire a separate non-contrast CT [41, 42]. For patients undergoing post-EVAR CTA follow-up to detect endoleak, use of a split-bolus injection technique has the potential to reduce radiation dose, by allowing acquisition of a simultaneous arterial and delayed phase. The split-bolus technique involves injecting two sequential CM boluses separated by a time delay (approximately 35 s), and acquiring a simultaneous arterial and venous phase CTA after the second CM bolus [78]. This can be further combined with DECT’s capability to reconstruct virtual non-contrast images, reducing the protocol down to a single CT acquisition, with significant radiation dose saving [79].

Performing a CTA prior to re-do cardiac surgery, with extra-cranial coverage to include the origin of the internal mammary arteries, allows identification of the location and can help surgeons alter surgical strategy, reducing the risk of intra-operative injury and improve outcomes [80, 81].

In thoracic aorta MRA (MR TA, Table 2), in addition to the routine MRA protocol, six to eight cine GRE short-axis slices can be acquired through the aortic valve, allowing characterisation of the aortic valve morphology (tricuspid/bicuspid/unicuspid), valve leaflet opening and coaptation. Using ECG-gating for the sagittal oblique ‘candy-cane’ CE-MRA helps combat aortic root motion (Fig. 6) [82]. ECG gating is not required for imaging the abdominal aorta. For combined MRA of the thoracic and abdominal aorta, for example in suspected aortitis, we perform separate gadolinium injections for each.

In patients in whom there is a clinical suspicion of large vessel vasculitis, or in cases of suspected aortic infection, acquisition of additional pre- and post-contrast high-resolution T1-weighted 3D spoiled GRE sequences with a fat selective prepulse allows an assessment for arterial mural enhancement (Aortitis, Table 2) (Fig. 7) [83, 84]. T1 double inversion recovery (DIR) ECG-gated, breath-held images provide excellent images of the aortic wall. DIR involves two successive 180° radiofrequency (RF) inversion pulses to null signal from moving blood in the aortic lumen, preserving the magnetisation of stationary tissues, and is excellent in delineating the structural anatomy of the aorta, particularly the aortic wall. Turbulence or slow flow within the lumen will result in incomplete nulling of the lumen, which can be difficult to distinguish from thrombus. Each slice requires a single breath-hold, which adds a considerable time penalty to the study when imaging of the entire aorta is required; therefore we recommend reserving DIRs for equivocal cases.

In cases of suspected aortic coarctation, additional phase contrast (PC) imaging is helpful (Coarctation, Table 2). This technique allows for a functional assessment of coarctation including quantification of peak gradient and collateral flow. PC is based on the principle that the spin phase of moving protons will change in proportion to their velocity. A bipolar magnetic gradient is applied to a volume of tissue; stationary spins will experience no net phase shift, but moving spins will experience a phase shift proportional to their velocity [85]. By applying a flow-sensitive PC sequence orthogonal to the direction of blood flow, flow can be quantified as either velocity or volume per unit time. PC imaging is performed at the site of the coarctation, immediately distal to the coarctation, and in the distal descending thoracic aorta immediately above the diaphragm. The severity of coarctation can be assessed by measuring the volume of collateral flow present, or by estimating the pressure gradient across the stenosis. The flow volume of the collateral circulation is calculated by subtracting the total flow volume in the proximal descending thoracic aorta immediately distal to the site of the coarctation from the volume in the distal descending thoracic aorta [86]. The percentage increase in flow volume from the collateral circulation increases linearly with the severity of stenosis at the coarctation site, and is the most useful measurement in the assessment of coarctation severity [87]. The PC acquisition through the site of maximal stenosis can be used to measure the peak velocity (v) across the site of coarctation and the maximal pressure gradient across the coarctation (ΔP) can then be measured by use of the modified Bernoulli equation (ΔP = 4v^2^) [86]. In patients with previous coarctation repair, the percentage increase in flow from the proximal to distal descending thoracic aorta is the most reliable indicator of haemodynamically significant restenosis [88].

### Mesenteric vessels

**Fig. 10:**
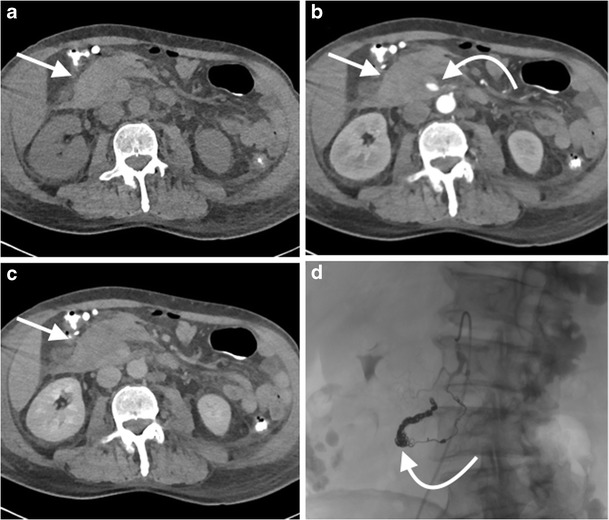
A 50-year-old man with oesophageal cancer was referred to CT following discovery of a mesenteric haematoma during exploratory laparoscopy. Axial non-contrast (**a**), arterial phase (**b**) and 70-s post contrast (**c**) images demonstrate an ill-defined mesenteric haematoma (*arrow*), a pancreaticoduodenal arcade pseudoaneurysm (*curved arrow*), with no active extravasation. The patient was taken to the interventional radiology suite and the pseudoaneurysm was occluded with coils; selected spot fluoroscopic image (**d**) demonstrates the occluded pancreaticoduodenal arcade filled with coils (*curved arrow*)

In the setting of suspected gastro-intestinal (GI) haemorrhage, endoscopy remains the initial test of choice, with CTA the imaging test of choice, reserved for those in whom endoscopy fails, or in the unstable patient with lower GI bleeding [89]. The same protocol used in post-EVAR assessment is suitable (CT post-EVAR, Table 1), and oral contrast should not be administered, as this reduces the ability to detect intraluminal haemorrhage [90]. CTA has a high diagnostic accuracy in detecting and localising the source of acute GI bleeding, both upper and lower, with a sensitivity of approximately 85% [91], and it can detect bleeding rates of as little as 0.3 ml/min (Fig. 10) [92]. Performing CTA prior to catheter angiography can increase the ability to successfully localise the bleeding source at catheter angiography [93]. DECT has the potential to improve the detection of active GI haemorrhage, with iodine map reconstructions providing additional diagnostic information regarding the presence and source of active bleeding [41].

**Fig. 11:**
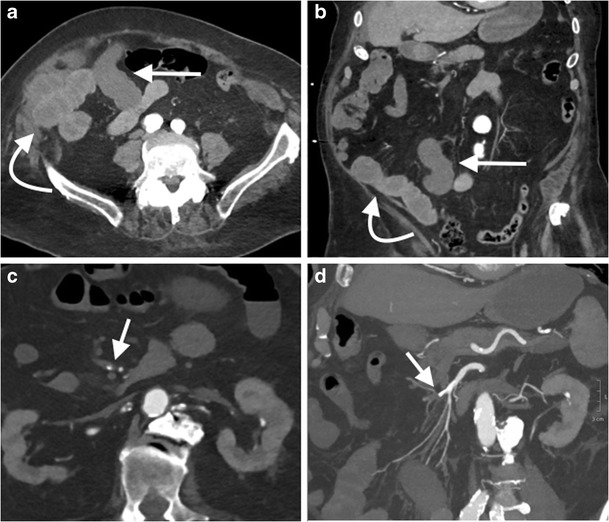
An 85-year-old woman developed severe abdominal pain two days post percutaneous aortic valve replacement and was referred for a CT mesenteric angiogram. Axial (**a**) and coronal (**b**) images from an arterial phase CT mesenteric angiogram demonstrate hypoenhancement of distal ileal loops (*straight arrow*) compared with adjacent proximal ileal and jejunal loops with normal mural enhancement (*curved arrow*). Axial (**c**) and coronal oblique images from the same study demonstrate focal occlusion of the mid-superior mesenteric artery with calcified plaque (*arrow*). Coronal oblique maximum intensity projection (MIP) reformat (**d**) is useful in demonstrating the point of SMA obstruction (*arrow*). Exploratory laparoscopy revealed extensive small bowel ischaemia, and the patient unfortunately expired

In patients with suspected acute mesenteric ischaemia, CTA is the first-line imaging test (CT mesenteric angiogram, Table 1) [94]. Whilst occlusive arterial disease is causative in 85% of cases, 15% are secondary to mesenteric venous thrombosis, which makes the acquisition of delayed venous phases helpful [95]. CT can also detect the ancillary findings of mesenteric ischaemia, such as bowel wall hypoenhancement, bowel wall thickening, fat stranding, pneumatosis intestinalis, portal venous gas, intra-peritoneal free gas and ascites (Fig. 11) [95, 96].

MRI is useful in the evaluation of chronic mesenteric ischaemia, and when CT is contraindicated (MRA mesenteric, Table 2). Mesenteric MRA has a high sensitivity and specificity in evaluating the proximal coeliac and superior mesenteric arteries [97], but is limited in its ability to detect distal mesenteric stenosis and occlusions compared to CTA [98].

### Renal vasculature

Dedicated renal vessel imaging is primarily performed in patients with suspected renovascular hypertension, and in the workup of potential donors and recipients in the live renal transplant program.

A standard renal CTA protocol (Table 1) consists of a single post-contrast bolus tracked arterial phase acquisition, with CTA performing well in the assessment of haemodynamically significant renal artery stenosis and fibromuscular dysplasia with high sensitivity and specificity [99–101]. Anatomic coverage should include the adrenals superiorly and extend inferiorly to the aortic bifurcation to exclude the presence of a pheochromocytoma arising from the adrenal medulla or an extra-adrenal paraganglioma, the most common site for which is the organ of Zuckerkandl, extra-adrenal chromaffin tissue near the origin of the inferior mesenteric artery [102].

**Fig. 12:**
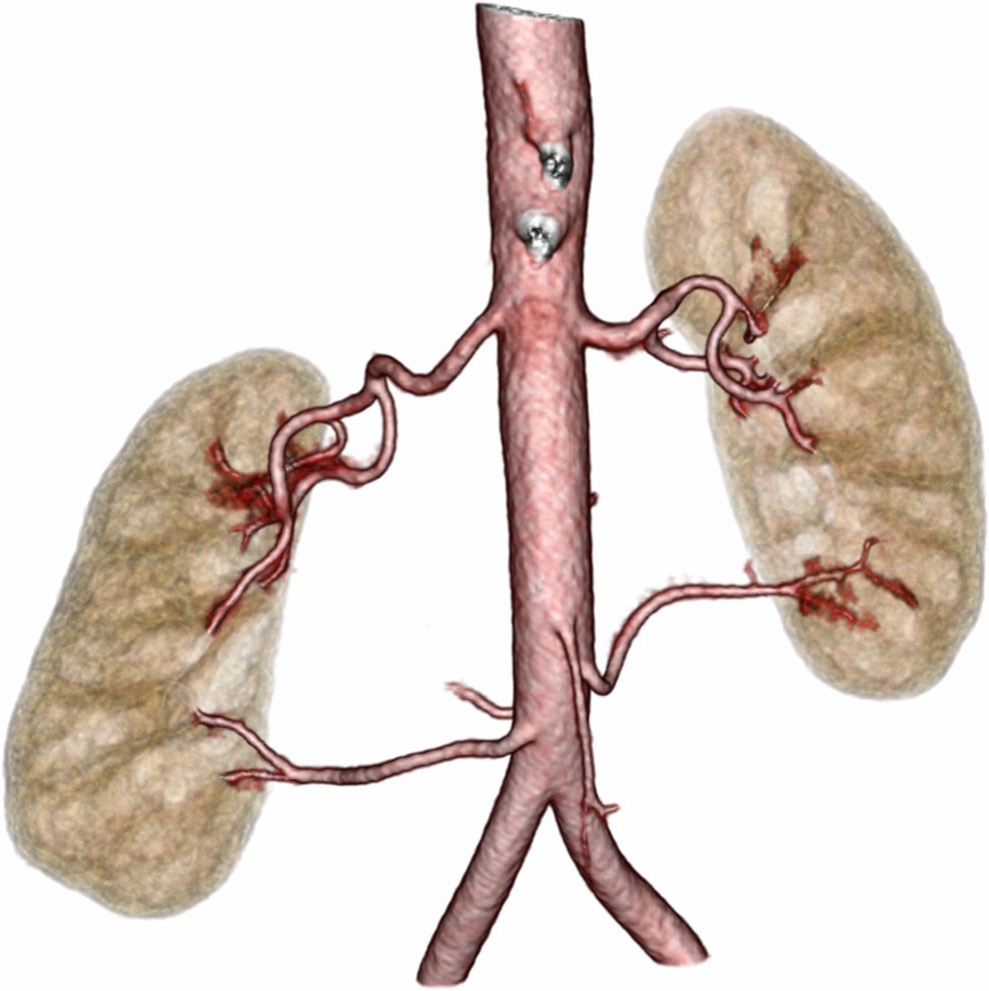
Three dimensional segmented volume rendered image of the kidneys and their arterial supply in a renal donor volunteer, segmented from an arterial phase renal artery angiogram CT, demonstrates bilateral accessory renal arteries supplying the lower renal poles

Laparoscopic living donor nephrectomy requires accurate pre-procedural vascular mapping (CT renal donor, Table 1). There is a large number of potential normal variants in renal arterial vascular anatomy. CT provides a better depiction of small renal arteries than MRI, and is the generally preferred modality [103]. CTA has a very high accuracy in identifying accessory renal arteries and pre-hilar arterial branching, which are important variants that the surgeon needs to be aware of (Fig. 12, ESM 4) [69, 104]. The non-contrast CT phase identifies renal calculi, with the arterial and nephrographic phases providing an accurate assessment of renal size, vascular anatomy and for any renal parenchymal lesions such as incidental renal tumours, an exclusion criterion for donors [105]. The urographic phase is used to evaluate for any anomalies of the renal collecting systems or ureters.

Although it does not offer the spatial resolution of CT, the lack of radiation makes renal artery CE-MRA (Table 2) an attractive modality, especially in younger patients. In a prospective comparison of 58 patients with suspected renovascular hypertension, Rountas et al. [99] found that CE-MRA has a slightly lower sensitivity than CTA for the detection of renal artery stenosis or fibromuscular dysplasia, 90% versus 94% respectively; this is likely due to the lower spatial resolution of MRI compared with CT.

For patients who cannot have gadolinium, a non-contrast renal MRA be performed with a respiratory-gated inflow balanced-SSFP sequence with inversion recovery saturation (examples: Inhance Inflow IR, General Electric; NATIVE TrueFISP, Siemens; B-TRANCE, Philips). These sequences accentuate the high signal of arterial blood by exploiting inflow-enhancement, similar to that used in time-of-flight (TOF) MRI [106]. Firstly, the volume of interest is saturated with a 180° radiofrequency pulse, inverting signal from background tissue and venous blood. Fresh, unsaturated arterial blood then flows into the slab, and a rapid 3D SSFP sequence is acquired after an appropriate inversion time to null signal from background tissue [107, 108]. This is a useful technique in patients suspected of having renovascular hypertension, with good agreement with CE-MRA and CTA for the presence of renal artery stenosis or fibromuscular dysplasia [109–112].

### Thoracic venous imaging

The most common reason for dedicated imaging of the thoracic venous circulation is in the evaluation of suspected superior vena cava (SVC) obstruction or thrombosis. Contrast-enhanced MR venography (CE-MRV) allows better differentiation of intraluminal thrombus from contrast mixing than CT venography (CTV), is equivalent to conventional venography in the assessment of central venous obstruction and is the non-invasive imaging modality of choice (CT SVC, Table 1; MR SVC, Table 2) [113, 114]. In patients who have difficulty with breath-holding, the post-contrast imaging can be performed free-breathing with respiratory gated navigator MRA [115].

### Abdominal and pelvic venous imaging

**Fig. 13:**
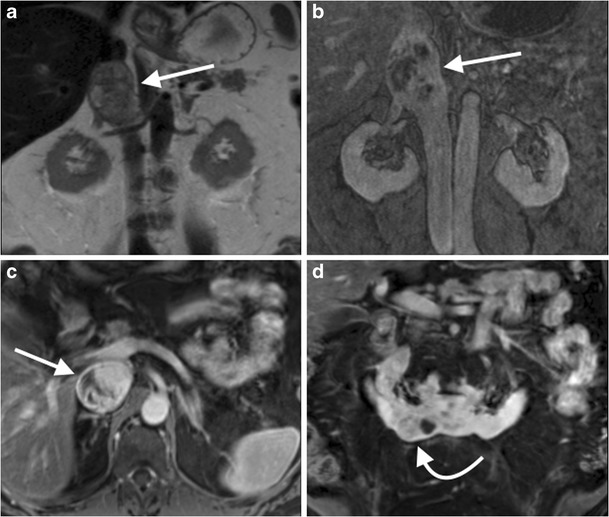
A 45-year-old man with bilateral leg swelling underwent an MR venogram of the abdomen and pelvis. Coronal image from a T2-weighted sequence (**a**) demonstrated an expanded suprarenal IVC with heterogeneous T2 high signal material, likely thrombus (*arrow*). Coronal (**b**) and axial (**c**) images from a venous phase T1-weighted fat-saturated post-gadolinium sequence demonstrates heterogeneous enhancement of the intraluminal IVC material, concerning for tumour thrombus. A coronal image from a T1-weighted fat-saturated post-gadolinium sequence of the abdomen (**d**) demonstrates a horseshoe kidney with an exophytic mass arising from the right lower moiety (*curved arrow*), which was subsequently confirmed as a papillary renal cell carcinoma on biopsy

**Fig. 14:**
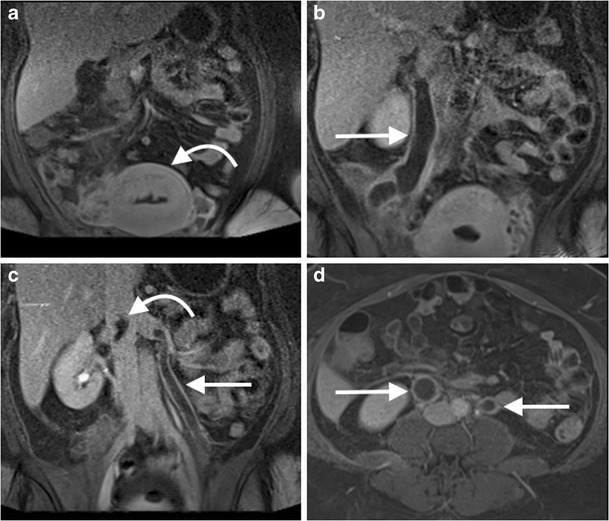
A 35-year-old woman underwent an abdominal MR venogram 5 days post dilatation and curettage for an intrauterine fetal death at 25 weeks. Coronal images from a post-contrast venous phase fat saturated T1 sequence of the abdomen demonstrates the post-partum uterus (**a**, *curved arrow*), with marked dilatation of the right (**b**, *arrow*) and left (**c**, *arrow*) gonadal veins with central filling defects consistent with bilateral gonadal vein thrombosis. The thrombus extends up to the juxtarenal IVC (**c**, *curved arrow*). Axial image from a T1 post-contrast venous phase fat saturated T1 sequence of the abdomen (**d**) demonstrates the expanded gonadal veins bilaterally with central filling defects

CT/MR venography of the abdomen and pelvis is commonly performed to assess for extension of lower limb deep vein thromboses (DVTs) or for a compressive venous syndrome, such as May-Thurner syndrome (obstruction of the left common iliac vein by the crossing right common iliac artery) [116]. MRV is preferable to CTV due to the presence of contrast mixing artefact with the latter, and imaging of the thighs can be included (Figs. 13 and 14). CTV is preferred when assessing potential inferior vena cava (IVC) filter complications, such as malposition, migration, tilting, caval thrombosis or perforation [117].

### Lower-limb angiography

CTA and MRA both provide a highly accurate vascular map, and have largely replaced catheter angiography in the diagnosis of lower limb ischaemia, acute and chronic [118]. A meta-analysis of the diagnostic performance of CTA and CE-MRA to detect haemodynamically significant arterial stenosis or occlusion demonstrated equal diagnostic accuracy of both techniques [119].

Acquiring a non-contrast CT phase allows for the assessment of calcified plaque, whilst the bolus-tracked CTA is highly accurate in depicting arterial occlusions and haemodynamically significant stenosis (CTA lower limbs, Table 1) [119–121]. This may be omitted in DECT systems with the capability to perform virtual non-contrast reconstructions [35]. The smaller below-the-knee runoff vessels are more challenging to interrogate due to their small size and difficulty opacifying adequately. Due to the speed of modern CT scanners, the CT often outruns the contrast bolus, affecting opacification of the below-the-knee arteries; acquiring an additional CTA phase with coverage from the knees through to the toes immediately after the bolus-tracked CTA can help assess these hard to image vessels. Providing separate reconstructions of each leg with a small field of view is also useful in improve spatial resolution.

CE-MRA provides an accurate luminal map of the arterial tree, with equal performance to CTA in the detection of arterial stenosis and occlusion (MRA lower limbs, Table 2) [119]. CE-MRA is often preferable to CTA, especially in patients with distal disease, and in those with heavily calcified vessels, which can hinder stenosis assessment on CTA [118]. MRA is limited in the assessment of stent patency due to local susceptibility artefact, where CTA is the preferred technique.

**Fig. 15:**
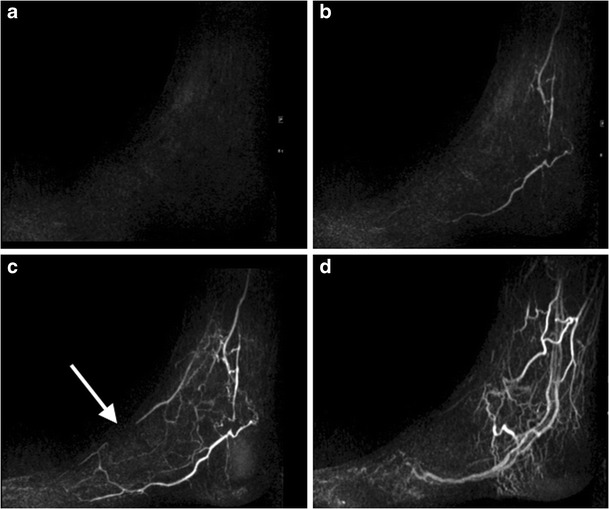
Selected sagittal images from a time-resolved MRA of the left foot in a patient with diabetes and foot pain. The initial mask image (**a**) demonstrates only a vague outline of the foot, with subsequent enhancement of the arteries (**b, c**), followed by venous filling at a later time point (**d**). This study demonstrates occlusion of the left dorsalis pedis in the left mid-foot (**c**, *arrow*), the likely cause of the patient symptoms

Time-resolved CE-MRA is an excellent method of assessing small vessel patency, and is particularly useful when imaging the below-the-knee vessels, where it is superior to standard CE-MRA [122, 123]. Both calves can be imaged simultaneously in the coronal plane following gadolinium injection, and when used, should be performed first in a lower limb CE-MRA. After this, a standard three-station (abdomen and pelvis, thighs, calves) bolus CE-MRA can be performed with a separate gadolinium injection. If foot arterial imaging is required, a separate time-resolved foot MRA is performed at the start of the examination before the calf MRA, using a boot coil with a sagittal acquisition separate gadolinium injection for the calves and for each foot (Fig. 15, ESM 5).

For patients with suspected popliteal artery entrapment syndrome, MRA is the preferred technique (MRA popliteal entrapment, Table 2). Typically, these are young patients, and definitive diagnosis requires imaging in multiple degrees of plantar and dorsiflexion. MRA allows precise analysis of local muscle anatomy, making it an ideal modality for diagnosis [124–126]. Time-resolved CE-MRA with the toes in the neutral position, in plantar flexion and then in dorsiflexion, with separate gadolinium injections for each.

### Upper limb angiography

**Fig. 16:**
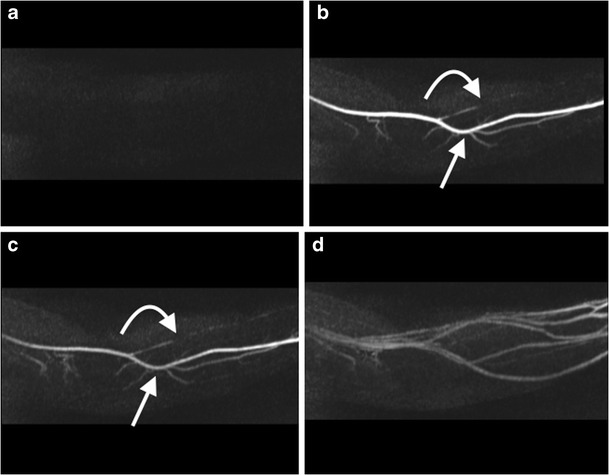
A 45-year-old man with a previous history of left radial artery thrombosis post coeliac artery stenting with persistent arm claudication. Time resolved MRA (**a-d**) of the left forearm demonstrates opacification of the left brachial artery, and left ulnar artery (*arrows*) with occlusion of the proximal left radial artery (*curved arrows*) (**b, c**), followed by venous filling (**d**)

Upper limb CTA and MRA is usually limited to one upper limb, and IV cannulation should be performed in the contralateral arm to avoid injection-related artefact. CTA is preferred in cases of suspected acute upper limb ischaemia due to its relatively quick time of acquisition, with MRA preferred in the chronic setting [127]. Where possible, upper limb CTAs should be acquired with the arm of interest raised above the head, with imaging extending from the aortic arch through the fingers (Upper extremity CTA, Table 1). A delayed CTA phase can help in assessment of the small arteries of the forearm, which may not be adequately opacified on the bolus-tracked CTA due to the scanner out-running the CM bolus. In upper extremity MRA (Table 2), time-resolved MRA is preferred for the forearm and hand vessels, with a standard CE-MRA for the proximal vessels (Fig. 16, ESM 6).

In cases of suspected thoracic outlet syndrome (TOS), MRA (Table 2) with positional manoeuvres is the preferred imaging modality for diagnosis, pre-operative planning and post-surgical follow-up [128, 129]. CE-MRA with coverage including the bilateral subclavian and axillary vessels is performed with the arms abducted approximately 150-160°, and repeated with the arms adducted, with separate contrast injections at each position.

### Pulmonary arteries

**Fig. 17:**
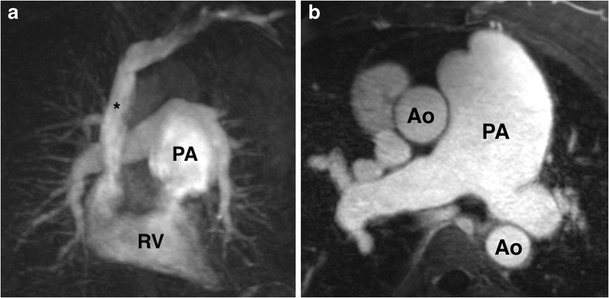
A pulmonary artery MRA in a 55-year-old woman with pulmonary hypertension. Single coronal image from time-resolved pulmonary artery MRA (**a**) and axial T1-weighted fat-saturated post-gadolinium angiogram image (**b**) demonstrate fusiform aneurysmal dilatation of the main pulmonary artery (*PA*)

Acute pulmonary embolism (PE) is the third most common acute cardiovascular disorder after myocardial infarction and stroke [130]. CT pulmonary angiography (CTPA) is the non-invasive reference standard for PE diagnosis and risk stratification, identifying adverse prognostic indicators such as right ventricular dilatation, interventricular septal bowing and a high embolus burden [131]. DECT, in particular iodine map reconstructions, have been shown to improve the accuracy of pulmonary emboli detection on CT, by demonstrating areas hypoperfused lung [43, 44]. MR pulmonary angiography (MRPA, Table 2) is a suitable alternative modality to diagnose PE in patients who cannot undergo CT, and is useful in the follow-up of pulmonary artery aneurysms (Fig. 17, ESM 7). In our experience, a time-resolved CE-MRA of the chest performed in the coronal plane with approximately nine phases yields diagnostic pulmonary arterial imaging in the majority of cases.

### Breast reconstruction flap planning

**Fig. 18:**
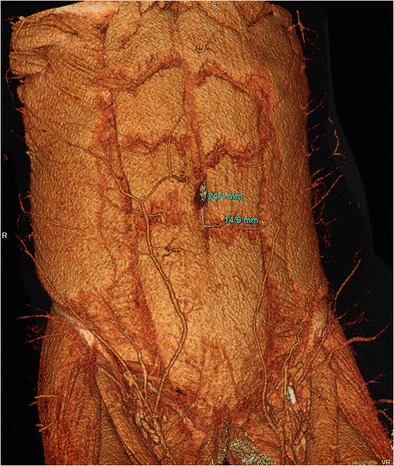
A 45-year-old woman with breast cancer undergoes a CT deep inferior epigastric artery perforator (DIEP) protocol prior to breast reconstruction. Coronal oblique 3D segmented VR image from the CT displays the abdominal wall musculature and superficial arterial supply. The location of the largest DIEP relative to the umbilicus is annotated with craniocaudal and mediolateral distance measurements to help the surgeon locate the vessel during surgery

The deep inferior epigastric perforator (DIEP) flap is the most common flap used in breast reconstruction. Raising a DIEP flap requires meticulous dissection of the DIEP vessels; however, there is significant heterogeneity in their branching pattern and location [132]. CTA is the current ‘gold standard’ for pre-operative vascular mapping, reducing operative time and postoperative complications [133–135]. A standard CT DIEP protocol is a single bolus-tracked CTA of the abdomen and pelvis, acquired in the caudo-cranial direction to mirror the direction of blood flow in the DIEP arteries (CT DIEP, Table 1). A segmented batch of 3D VR images displaying the number and site of DIEPs relative to the rectus sheath and overlying skin, providing the distance of the largest perforator from the umbilicus, is of particular use in surgical planning (Fig. 18, ESM 8). CTA is also used in patients undergoing pre-operative assessment for a superior gluteal artery perforator (SGAP) flap (Table 1); in this protocol, the patient is scanned prone, with anatomical coverage from the umbilicus to the mid-thighs.

## Conclusions

A basic understanding of the technical, physiological and pathological challenges posed by vascular imaging allows the creation of bespoke, safe imaging protocols that can help improve diagnosis and impact outcomes. The challenges posed by cardiovascular imaging will continue to drive technological improvements in scanner technology, and it is important that radiologists continue to improve and streamline vascular imaging practices.

## Electronic supplementary material

ESM 1 Video of rotating 3D segmented VR fusiform ascending thoracic aortic aneurysm (MP4 1389 kb)

ESM 2 Video of rotating 3D segmented VR thoracic aorta (MP4 1049 kb)

ESM 3 Video of rotating 3D segmented VR abdominal aorta (MP4 620 kb)

ESM 4 Rotating video of 3D segmented VR image of kidneys and their arterial supply (MP4 8779 kb)

ESM 5 Video of time-resolved MRA of the left foot (MOV 413 kb)

ESM 6 Video of time-resolved MRA of the left forearm (MOV 274 kb)

ESM 7 Cine clip of time-resolved pulmonary artery MRA. *Ao* aorta, *RV* right ventricle (MOV 998 kb)

ESM 8 Rotating video of 3D segmented VR image of DIEP vessels, abdominal wall musculature, and overlying skin (MP4 15,292 kb)
